# Anti-reflux mucosectomy for proton pump inhibitor refractory gastroesophageal reflux disease with hiatal hernia: A case report

**DOI:** 10.1097/MD.0000000000041166

**Published:** 2025-02-21

**Authors:** Ying Ba, Sheng Liu, Ning Shi, Yan Chen, Hongwei Xu

**Affiliations:** a Department of Gastroenterology, Shandong Provincial Hospital, Shandong University, Jinan, China; b Department of Gastroenterology, Binzhou Medical University Hospital, Binzhou, China; c Department of Neurosurgery, Binzhou Medical University Hospital, Binzhou, China.

**Keywords:** 24h pH impedance monitoring, anti-reflux mucosectomy (AMRS), gastroesophageal reflux disease (GERD), hiatus hernia (HH), high resolution esophageal manometry (HREM), proton pump inhibitor refractory

## Abstract

**Introduction:**

Gastroesophageal reflux disease (GERD) is a prevalent chronic condition primarily treated with proton pump inhibitors (PPIs). However, patients with PPI-refractory GERD and hiatal hernia present significant therapeutic challenges, with conventional treatments often yielding unsatisfactory outcomes. While anti-reflux mucosectomy (ARMS) has emerged as a promising treatment, its efficacy in cases with hiatal hernia remains understudied. The primary objective of this case report is to evaluate the safety and efficacy of ARMS in treating PPI-refractory GERD complicated by hiatal hernia.

**Case presentation:**

We report the case of a 77-year-old female patient with PPI-refractory GERD and significant hiatal hernia who underwent ARMS. The procedure successfully reconstructed the barrier at the gastroesophageal junction (GEJ), resulting in marked symptom improvement and enhanced quality of life at 3-month follow-up.

**Conclusions:**

This case demonstrates that ARMS can be an effective therapeutic option for PPI-refractory GERD patients with hiatal hernia, challenging previous contraindications. Further studies with larger patient cohorts are warranted to validate these findings.

## 1. Introduction

Proton pump inhibitor (PPI) refractory gastroesophageal reflux disease (rGERD) is defined as the presence of persistent gastroesophageal reflux disease (GERD) symptoms despite the optimization of PPI therapy. Anti-reflux mucosectomy (ARMS) is a relatively new endoscopic procedure for resection (EMR) or endoscopic submucosal dissection (ESD) performed around the gastroesophageal junction (GEJ), which is considered to be an effective treatment for rGERD.^[1–3]^ However, previous studies have suggested that ARMS was contraindicated in rGERD patients with hiatal hernia.^[2,4]^ Nevertheless, recent studies have shown that ARMS may be an effective anti-reflux procedure in rGERD patients with moderate hiatal hernia.^[5]^ In this case report, we present a case of rGERD with hiatal hernia treated with ARMS, which resulted in a favorable prognosis. Our findings suggest that ARMS is an effective treatment option for rGERD patients with hiatal hernia.

## 2. Case presentation

A 77-year-old woman was admitted to our department due to a long-lasting problem of acid reflux and serious heartburn that has been going on for 8 years. Over the past month, her symptoms have worsened, including persistent acid reflux, heartburn, and intermittent vomiting of food, particularly at night. Despite trying various medications, such as different types of proton pump inhibitors and prokinetics, none have provided relief. The patient’s medical history includes degeneration of the lumbar vertebrae. There is no family history of similar conditions, no prior surgical interventions or traumatic events, and she does not smoke or consume alcohol. The physical examination showed no significant findings. The laboratory results were mostly normal, except for mild hypoalbuminemia (36.5 g/L). Chest radiography revealed mild pneumonia, fibrous foci in both lungs, calcification of the aortic and coronary arteries, hiatal hernia, thickening of the lower esophagus, and slightly enlarged surrounding lymph nodes.

**Figure 1.**
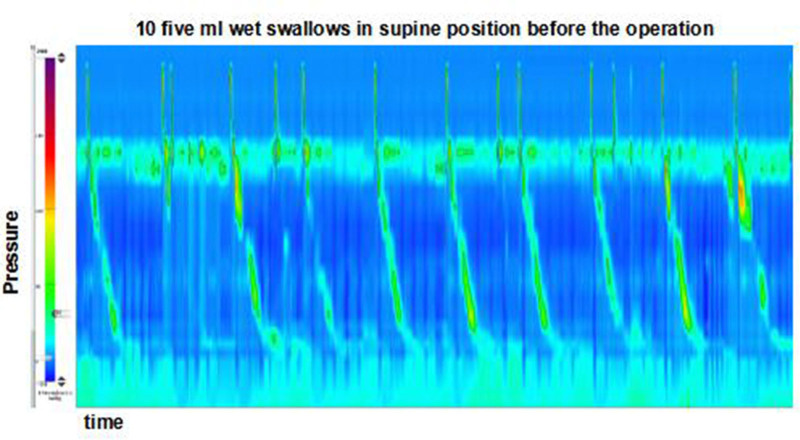
Preoperative high resolution esophageal manometry (HREM) shows a weak esophageal peristalsis and a low pressure of LES. HREM = high resolution esophageal manometry, LES = lower esophageal sphincter.

**Figure 2.**
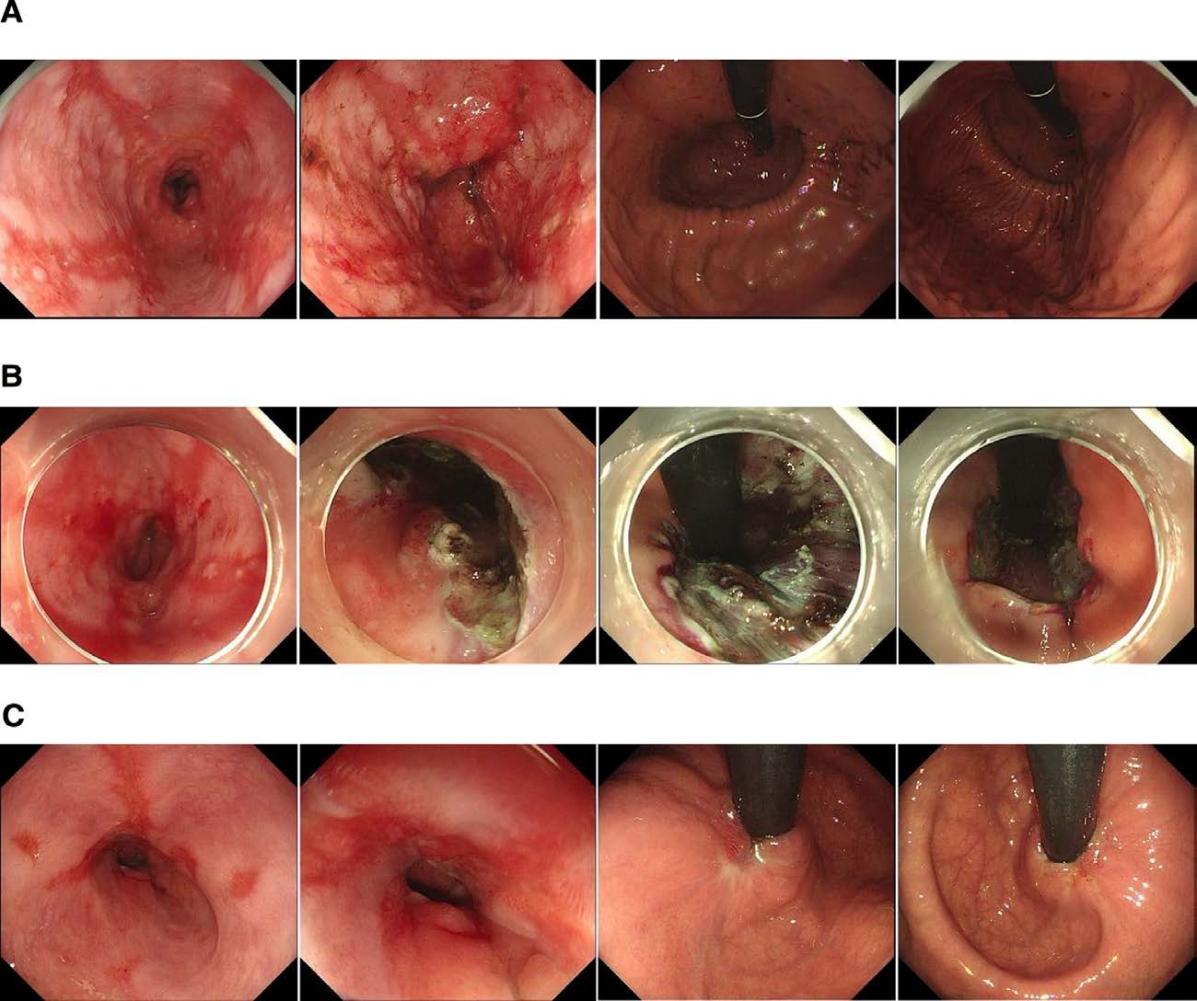
Gastroscopy conducted prior to ARMS revealed severe reflux esophagitis (LA grade D) and hiatus hernia. (A) The ARMS procedure was performed using the ESD method. (B) Following the ARMS procedure, a subsequent gastroscopy showed an improvement in erosive esophagitis and the gastroesophageal flap valve was graded as I (C). ARMS = anti-reflux mucosectomy, ESD = endoscopic submucosal dissection.

High-resolution esophageal manometry revealed a low pressure of the lower esophageal sphincter (LES) and a normal median integrated relaxation pressure (IRP) of 4 seconds. The analysis also indicated a separation of 2.6 cm between the LES and crural diaphragm (CD), consistent with type II esophagogastric junction (EGJ) morphology (Fig. 1). Ten 5-mL water swallows were performed in the supine position, of which 6 had weak contractions and 4 had failed contractions, according to the Chicago Classification V4.0.^[6]^ The mean distal contractile integral was 146 mm Hg s cm (Table 1). Gastroscopy was conducted to rule out high-grade neoplasia and carcinoma in the upper gastrointestinal tract, particularly the esophagus. The results revealed severe reflux esophagitis (LA grade D) with bleeding in the distal esophagus, as well as a hiatus hernia. The gastroesophageal flap valve was classified as grade IV according to the Hill classification.^[7,8]^ The distance between the squamous columnar junction and the impression of the diaphragm was approximately 3 cm (Fig. 2). The lower esophageal biopsy showed acute and chronic inflammation of the gastroesophageal junction mucosal tissue, squamous hyperplasia, the presence of lymphatic follicles in the lamina propria, and atrophic inflammation of the gastric mucosa with intestinal metaplasia. The preoperative scores on the GERD questionnaire (GERD Q) and GERD health-related quality of life (GERD-HRQL) were 12 and 25, respectively.

**Table 1:** High resolution manometry (HRM) was employed to assess pre- and postoperative esophageal peristalsis.

	LES pressure (mm Hg)	4sIRP (mm Hg)	DCI (mm Hg s cm)	Mean DCI (mm Hg s cm)
Preoperation	−12.9	−10.2	47	146
			0	
			204	
			12	
			129	
			325	
			193	
			45	
			399	
			100	
Postoperation	8.1	−1.9	606	445
			180	
			25	
			644	
			24	
			484	
			389	
			709	
			0	
			1391	

DCI = distal contractile integral, HRM = high resolution manometry, IRP = integrated relaxation pressure, LES = lower esophageal sphincter.

Due to the presence of severe erosion and bleeding in the lower esophagus, the following prescription was given for a period of 6 weeks: esomeprazole orally twice a day with a dosage of 40 mg each time, combined with hydrotalcid taken 3 times a day with a dosage of 1 g each time. After completing the 6-week medication treatment, the patient underwent anti-reflux mucosectomy (ARMS) smoothly. ARMS of the esophagogastric junctional (EGJ) mucosa was performed using either endoscopic mucosal resection (EMR) or ESD, with a minimum length of 3 cm (1 cm in the esophagus and 2 cm in the stomach). A 270-degree mucosal resection was conducted along the side of the lesser curvature of the stomach, while preserving a sharp mucosal valve at the gastric cardia.^[4]^ The ARMS procedure using the ESD method was performed by 2 experienced endoscopists, using a single-channel endoscope (GIF-J260; Olympus Co., Ltd.). The patient was under conscious sedation with intravenous propofol. Initially, dots were marked beyond the pre-resection region using argon plasma coagulation. Subsequently, a solution consisting of 0.9% saline with a small amount of epinephrine and indigo carmine was injected into the submucosal layer to facilitate dissection of the lesion mucosa. Following submucosal injection, a complete circumferential mucosal incision was made based on the marked dots. Finally, dissection of the submucosal tissue was performed, achieving en bloc resection without any adverse events (Fig. 2). The entire operation lasted for 1 hour.

**Figure 3.**
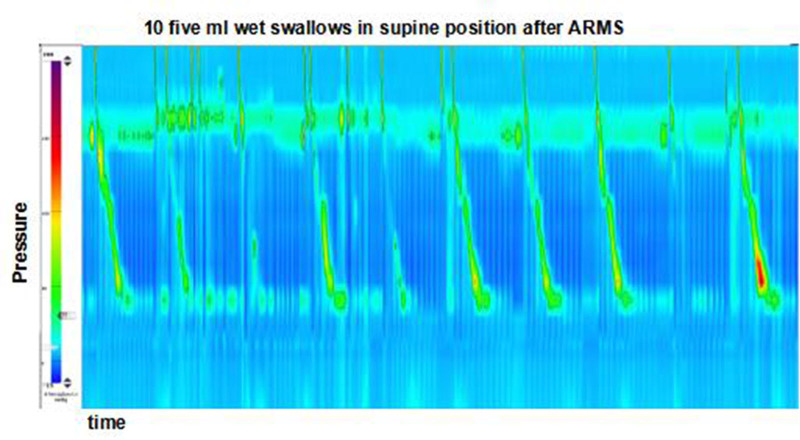
Postoperative high resolution esophageal manometry (HREM) shows an enhancement of esophageal peristalsis. ARMS = anti-reflux mucosectomy, HREM = high resolution esophageal manometry.

The patient initiated water intake on the first day, followed by a fluid diet on the second day. Starting from the third day until 1 week after the operation, the patient transitioned to a soft food diet. It was recommended for the patient to take PPI orally for 8 weeks after ARMS. A series of follow-up studies were conducted 3 months after the procedure. The patient did not experience dysphagia and reflux symptoms significantly improved without the need for PPI therapy. High-resolution esophageal manometry revealed that the pressure of the LES was still below normal (−12.9 vs 8.1 mm Hg), and the median IRP for a 4-second duration was higher than preoperation (−10.2 vs −1.9 mm Hg), although still within the normal range, indicating the absence of dysphagia. Analysis of water swallows showed enhanced esophageal peristalsis, with 2 weak contractions and 3 failed contractions out of ten 5-mL water swallows (Fig. 3, Table 1). This improvement may be attributed to the reduction of esophageal mucosa inflammation and the recovery of peristalsis in the esophagus. Gastroscopy revealed an improvement in erosive esophagitis from grade D to grade A, while the hiatus hernia remained unchanged. The gastroscope passed through the cardia without any apparent resistance. The gastroesophageal flap valve was classified as grade I (Fig. 2). The postoperative GERD questionnaire (GERD Q) score was 8, and the GERD patient health-related quality of life (GERD-HRQL) score was 12.

## 3. Discussion and conclusions

Our case demonstrates significant clinical improvement following ARMS in a patient with PPI-refractory GERD and hiatal hernia, with multiple objective and subjective measures supporting treatment efficacy. Key findings include marked improvement in symptom scores (GERD-Q score reduction from 12 to 8 and GERD-HRQL score decrease from 25 to 12), endoscopic healing of erosive esophagitis from LA grade D to grade A, and enhanced esophageal motility parameters on manometry. The procedure was completed without immediate complications, and the patient successfully discontinued PPI therapy at 3 months postprocedure. Notable improvements were also observed in the gastroesophageal flap valve grade (from IV to I) and esophageal peristalsis, with a reduction in failed contractions from 4 to 3 out of 10 swallows. While the hiatal hernia remained unchanged, the reconstruction of the mucosal flap valve appeared to provide adequate reflux control. These comprehensive improvements across multiple parameters suggest that ARMS can effectively address both the anatomical and functional aspects of refractory GERD, even in the presence of hiatal hernia. Our findings are particularly relevant given that hiatal hernia has traditionally been considered a relative contraindication for ARMS, and they suggest potential expansion of the procedure’s indications in carefully selected patients.

The prevalence of GERD has seen an increase in recent years, with varying incidence rates from country to country. A recent epidemiological survey reported a prevalence of GERD ranging from 2.5% to 7.8% among Chinese individuals.^[9]^ PPI is considered the first-line pharmacologic therapy for GERD, although up to 40% of patients do not respond to PPI treatment.^[10]^ The anti-reflux barrier, which consists of the LES and the diaphragm, along with the His angle between the fundus of the stomach and the esophagus, also plays a role in preventing gastroesophageal reflux. Low pressure in the LES and the presence of a hiatus hernia can contribute to increased reflux burden and acid exposure. There are several potential therapies available for GERD patients with hiatus hernia, and their efficacy can vary. These include laparoscopic fundoplication, stretta, transoral incisionless fundoplication (TIF), medigus ultrasonic surgical stapler (MUSE), magnetic sphincter augmentation, and anti-reflux mucosectomy (ARMS). The main goal of these procedures is to restore the physical barrier at the esophagogastric junction (EGJ) to resist reflux. Laparoscopic fundoplication is the recommended choice, with approximately 65.3% of patients experiencing improvement in symptoms. The most commonly reported complications associated with laparoscopic procedures include gastric or esophageal injury, bleeding, pneumonia, bloating, and dysphagia.^[11]^

Anti-reflux mucosectomy (ARMS) was first reported by Hitoshi in 2003. Hitoshi operated on a patient with Barrett’s esophagus and high-grade intraepithelial dysplasia using GEJ EMR, and the patient experienced an improvement in GERD symptoms.^[12]^ The authors then refined the endoscopic procedure and published a case series in 2014, demonstrating technical success in all patients and symptomatic improvement.^[4]^ They concluded that patients with PPI refractory GERD without a sliding hiatal hernia are the best candidates for ARMS treatment. Another retrospective review selected 19 patients with medically refractory GERD and short segment (shorter than 2.0 cm in length) hiatal hernia (SSHH) to evaluate the procedure.^[1,13]^ The study found that two-thirds of patients were able to discontinue their PPI. The most frequent indication for antireflux surgery has been severe GERD unresponsive or partially responsive to medical therapy. Patients who fail to respond to PPI should undergo tests to determine the reliability of GERD diagnosis. These examinations include upper endoscopy, esophageal manometry, 24h pH impedance monitoring, and gastric emptying studies if symptoms of bloating are prominent. A meta-analysis showed that the rate of complete clinical response was 65.3%. The most common complications are dysphagia and bleeding, with rates of 11.4% and 5.0% respectively.^[14]^

ARMS is a novel endoscopic anti-reflux surgery aimed at reconstructing the mucosal flap valve at the gastric cardia. Currently, there are no universally accepted indications for ARMS. A study conducted by Haruhiro Inoue suggests that patients with PPI refractory GERD, without a sliding hiatal hernia and possibly short-segment Barrett’s esophagus, appear to be the most suitable candidates for ARMS treatment. Another article suggests that hiatus hernias larger than 3 cm should be excluded.^[4,15]^ In our study, we aimed to investigate the effectiveness of ARMS treatment in patients with significant hiatus hernia. A comprehensive review of the literature indicates that ARMS therapy has a positive effect on rGERD. Since the primary objective of ARMS surgery is to reconstruct the GEJ barrier, we believe that the presence of Hiatus Hernia should not be considered a contraindication.

The reported case in our study involves a patient with PPI refractory GERD, a significant hiatus hernia, advanced age, and a low willingness to undergo surgery. During the postoperative follow-up, no complications such as dysphagia were observed, and the patient discontinued PPI medication 3 months after the operation due to well-controlled symptoms. Postoperative examinations, including manometry and gastroscope, showed improvement, leading to a marked enhancement in the patient’s quality of life. ARMS offers several advantages over other treatments, including high security, shorter hospital stay, and lower cost. It can be performed by an ordinary doctor without requiring special equipment, and it does not involve the placement of magnetic beads or patches in the body. This treatment is particularly suitable for patients with Barrett’s esophagus and intraepithelial neoplasia as it addresses both reflux and mucous membrane pathological changes simultaneously. In case of postoperative complications such as dysphagia, endoscopic dilation can be performed to resolve the issue. If ARMS fails, laparoscopic fundoplication remains an alternative option. However, as a new surgical method, ARMS has its limitations. There is a scarcity of cases and a lack of large-sample multi-center studies, making the long-term effectiveness uncertain. Additionally, there is no consensus on postoperative management, and a unified standard for evaluating postoperative effectiveness is lacking. The degree of postoperative scar formation can be influenced by intraoperative manipulation, postoperative management, and patient constitution, leading to variations in surgical outcomes. It is crucial for an experienced operator to develop an individualized treatment plan for each patient, striking a balance between optimal anti-reflux effects and postoperative dysphagia.

While our case report demonstrates promising results, several important limitations warrant discussion. As a single case study, our findings, although encouraging, cannot be generalized to the broader GERD patient population, and the successful outcome may be influenced by patient-specific factors not representative of all GERD cases with hiatal hernia. The 3-month follow-up period, while showing positive results, may be insufficient to assess long-term outcomes, including the durability of the anti-reflux effect and potential late complications. The natural history of scar formation and its impact on symptom control requires extended observation. Additionally, the lack of standardized outcome measures for ARMS procedures poses challenges in comparing results with other treatment modalities, and the absence of postprocedure pH impedance monitoring limits our ability to quantify the actual reduction in reflux episodes. Technical considerations also present limitations, as the procedure’s success may be operator-dependent, requiring significant endoscopic expertise, and the learning curve for ARMS in patients with hiatal hernia may be steeper than in standard cases. Furthermore, the cost-effectiveness of ARMS compared to alternative treatments was not evaluated, and the procedure’s accessibility may be limited by the need for specialized endoscopic expertise and equipment. These limitations highlight the need for larger, prospective multicenter studies with longer follow-up periods, standardized protocols for patient selection and outcome assessment, cost-effectiveness analyses comparing ARMS with other treatment options, and development of technical guidelines specific to cases with hiatal hernia. Further research addressing these limitations and investigating predictive factors for treatment success will help establish the optimal role of ARMS in the management of PPI-refractory GERD with hiatal hernia. Understanding these limitations is crucial for both clinicians considering ARMS as a treatment option and researchers planning future studies in this field.

In conclusion, our case report suggests that the use of ARMS is safe and effective in GERD patients with significant hiatal hernia. However, to obtain more robust evidence, our center is currently conducting a follow-up study with a larger sample size.

## Author contributions

**Conceptualization:** Ying Ba.

**Data curation:** Ying Ba, Sheng Liu, Ning Shi, Yan Chen.

**Formal analysis:** Sheng Liu, Ning Shi, Yan Chen.

**Investigation:** Ying Ba, Sheng Liu, Ning Shi, Yan Chen.

**Project administration:** Ying Ba, Hongwei Xu.

**Writing – original draft:** Ying Ba.

**Writing – review & editing:** Hongwei Xu.
